# Organism-specific differences in the binding of ketoprofen to serum albumin

**DOI:** 10.1107/S2052252522006820

**Published:** 2022-07-16

**Authors:** Mateusz P. Czub, Alan J. Stewart, Ivan G. Shabalin, Wladek Minor

**Affiliations:** a Department of Molecular Physiology and Biological Physics, University of Virginia, 1340 Jefferson Park Avenue, Charlottesville, VA 22908, USA; b Center for Structural Genomics of Infectious Diseases (CSGID), University of Virginia, 1340 Jefferson Park Avenue, Charlottesville, VA 22908, USA; c School of Medicine, University of St Andrews, St Andrews KY16 9TF, United Kingdom; Chinese Academy of Sciences, China

**Keywords:** organism-dependent studies, drug interactions, drug transport, human serum albumin, NSAIDs, anti-inflammatory drugs, ketoprofen

## Abstract

Serum albumin is a circulatory transport protein that has a highly conserved sequence and structure across mammalian organisms. Its ligand-binding properties are of importance as albumin regulates the pharmacokinetics of many drugs. Due to the high degree of structural conservation between mammalian albumins, nonhuman albumins such as bovine serum albumin or animal models are often used to understand human albumin–drug interactions. Ketoprofen is a popular nonsteroidal anti-inflammatory drug that is transported by albumin. Here, it is revealed that ketoprofen exhibits different binding-site preferences when interacting with human serum albumin compared with other mammalian albumins, despite the conservation of binding sites across species. The reasons for the observed differences were explored, including identifying ketoprofen binding determinants at specific sites and the influence of fatty acids and other ligands on drug binding. The presented results reveal that the drug-binding properties of albumins cannot easily be predicted based only on a complex of albumin from another organism and the conservation of drug sites between species. This work shows that understanding organism-dependent differences is essential for assessing the suitability of particular albumins for structural or biochemical studies.

## Abstract 2

Ketoprofen exhibits different binding-site preferences when interacting with mammalian albumins from different species. The drug-binding properties of albumins cannot easily be predicted based only on a complex of albumin from another species even when the drug-binding sites exhibit a high degree of conservation between species.

## 1. Introduction

Serum albumin (SA) is the most abundant protein in mammalian blood plasma, with a concentration in human plasma of between 35 and 50 g l^−1^ (Doweiko & Nompleggi, 1991 ▸). Structurally, albumin consists of three homologous domains containing multiple binding pockets that accommodate various classes of small molecules. Albumin serves as a fatty-acid, metal-ion and drug transporter in the blood (Peters, 1995 ▸; Lombardo *et al.*, 2018 ▸). More than 600 FDA-approved drugs have been reported to bind to plasma proteins, with albumin being the main binding protein, at levels where ≥50% of the total drug concentration present is bound (Lombardo *et al.*, 2018 ▸). However, only about 30 FDA-approved drugs have structures of albumin complexes available in the Protein Data Bank (PDB), including 25 drugs in complex with human serum albumin (HSA). Ten binding sites within albumin have been characterized as drug sites, and nine of these have been demonstrated to bind at least three FDA-approved drugs (Czub *et al.*, 2020 ▸).

The binding of drugs to plasma proteins is routinely evaluated during drug-lead optimization (Bohnert & Gan, 2013 ▸; Trainor, 2007 ▸). Because of its high concentration in the blood, drug binding to albumin is a significant factor that determines the efficacy of many drugs. Albumin acts as both a transporter and reservoir, delivering drugs to their sites of action, extending their circulatory half-life and reducing aggregation and other unwanted effects. However, strong binding of a drug to albumin can negatively affect its availability in the body. According to the free-drug theory, only unbound drug molecules are expected to be pharmacologically active (Bohnert & Gan, 2013 ▸; Trainor, 2007 ▸). Due to the high conservation of its sequence and structure (Supplementary Fig. S1), the drug-binding properties of albumin are typically expected to be similar across mammals. Nevertheless, significant differences in the effects of some drugs in humans compared with other animals have been reported, and it has been suggested that this may be due to differences in albumin binding [for example valproate in mice (Kosa *et al.*, 1997 ▸; Acharya *et al.*, 2006 ▸) and cefotetan in rats (Colclough *et al.*, 2014 ▸)]. However, structural confirmation of such differences across species is currently lacking. To date, naproxen (a nonsteroidal anti-inflammatory drug) is the only drug whose binding to SAs from various sources has been structurally studied and discussed (Bujacz *et al.*, 2014 ▸). It was reported that binding of naproxen to SAs from different species is partially conserved, as it binds to drug sites 2 and 7 in equine serum albumin (ESA), bovine serum albumin (BSA) and leporine serum albumin (LSA), but additionally to drug site 6 in LSA and drug site 1 in BSA. In the case of HSA, naproxen was reported to only bind to drug site 3.

**Figure 1:**
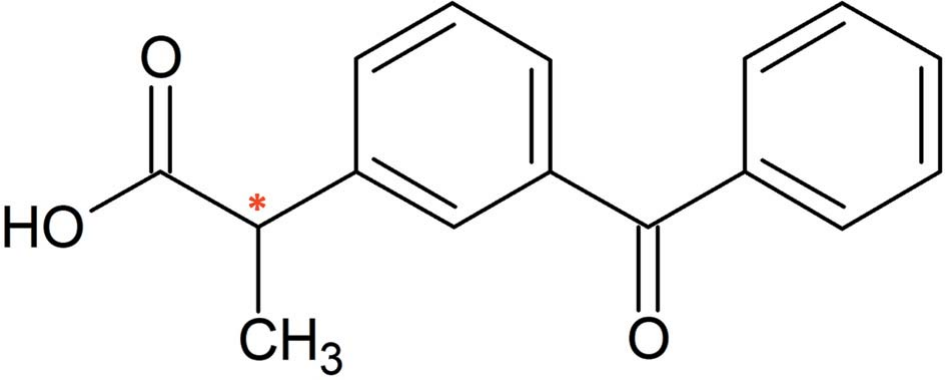
Chemical structure of ketoprofen; the chiral center is labeled with an asterisk.

Ketoprofen is a nonsteroidal anti-inflammatory drug (NSAID) that is used to treat rheumatoid arthritis, osteo­arthritis and dysmenorrhea, and to alleviate moderate pain, primarily in humans (Gallelli *et al.*, 2007 ▸). It is also used to treat domesticated animals such as cats, dogs and horses (Lees *et al.*, 2003 ▸; Hazewinkel *et al.*, 2003 ▸; Owens *et al.*, 1995 ▸). Ketoprofen possesses a chiral center and is typically administered orally or topically in the form of a racemic mixture; its structure is shown in Fig. 1 ▸. The (*S*)-enantiomer is primarily responsible for inhibiting prostaglandin synthesis, while the (*R*)-enantiomer is responsible for its analgesic activity (Cooper *et al.*, 1998 ▸; Ghezzi *et al.*, 1998 ▸). However, it has been observed that a fraction of (*R*)-ketoprofen undergoes metabolic conversion to the (*S*)-enantiomer in patients (Lorier *et al.*, 2016 ▸). Ketoprofen is a commonly used over-the-counter NSAID, but multiple side effects have been reported for this drug (Kantor, 1986 ▸; Le Loet, 1989 ▸). Moreover, at the time of writing this article the drugs.com database (https://www.drugs.com/) lists ketoprofen to have 70 major and 262 moderate interactions with other drugs. The side effects of ketoprofen and its interactions with other medications make it an undesirable analgesic for some patients.

About 99% of ketoprofen in human plasma is bound to albumin (Verbeeck *et al.*, 1983 ▸). Ketoprofen binding to mammalian albumins has been extensively studied using equilibrium dialysis (Dubois *et al.*, 1993 ▸), calorimetry (Zielinski *et al.*, 2020 ▸; Misra & Kishore, 2013 ▸) and spectroscopic methods (Maciążek-Jurczyk, 2014 ▸; Bi *et al.*, 2011 ▸). These studies reported relatively similar ketoprofen binding affinities for HSA (*K*_d_ = 85 µ*M* at 288.15 K; Bi *et al.*, 2011 ▸), BSA (*K*_d1_ = 30 µ*M*, *K*_d2_ = 189 µ*M*; Misra & Kishore, 2013 ▸) and LSA (*K*_d_ = 49 µ*M*; Zielinski *et al.*, 2020 ▸). Recently, a number of structures of ketoprofen complexes with BSA (Castagna *et al.*, 2019 ▸), ESA (Czub *et al.*, 2020 ▸) and LSA (Zielinski *et al.*, 2020 ▸) have been determined by X-ray crystallography. Accurate knowledge of the locations of ketoprofen binding sites on HSA would allow a better understanding of the factors that influence its circulatory transport in humans, including the effects of non-enzymatic glycation caused by diabetes (Anguizola *et al.*, 2013 ▸) and competition with other drugs for specific binding sites that may cause displacement (Czub *et al.*, 2020 ▸; Bohnert *et al.*, 2010 ▸). Here, we present the first crystal structure of HSA in complex with ketoprofen, which provides insights into the molecular basis of its circulatory transport. We also compare the ketoprofen binding modes observed in other mammalian albumins and discuss potential reasons for the observed interspecies differences, which have implications for albumin–drug studies.

## 2. Materials and methods

### 2.1. Materials

Recombinant HSA expressed in *Pichia pastoris* was purchased from Sigma–Aldrich, St Louis, Missouri, USA (catalog No. A7736; ≥90% purity) as a lyophilized powder and was purified further as described below. According to the vendor, the construct has a single deletion of Asp from the N-terminus (Asp1) to create a hypoallergenic construct by eliminating the principal copper- and nickel-binding site of albumin. In addition, Cys34 was blocked by adding free cysteine to improve stability, monomer content and homogeneity. Ketoprofen was purchased from Santa Cruz Biotechnology, Dallas, Texas, USA (catalog No. 205359; ≥99% purity) in the form of a racemic mixture, which represents the commercially available formulation of this drug.

### 2.2. Protein purification for crystallization

HSA was dissolved in a buffer consisting of 50 m*M* Tris, 20 m*M* NaCl pH 7.4 and subjected to gel filtration using the same buffer on a Superdex 200 column attached to an ÄKTA FPLC (GE Healthcare, Chicago, Illinois, USA) at 4°C. The HSA concentration was estimated spectrophotometrically by measuring the absorbance at 280 nm with a Nanodrop 2000 (Thermo Scientific, Waltham, Massachusetts, USA) using an extinction coefficient ɛ_280-HSA_ of 34 440 *M* ^−1^ cm^−1^ and a molecular weight MW_HSA_ of 66 470 kDa. Collected fractions of monomeric HSA were combined and concentrated to 162 mg ml^−1^ using an Amicon Ultra Centrifugal Filter with a 30 kDa molecular-weight cutoff (Sigma, catalog No. UFC903024).

### 2.3. Protein crystallization

Crystallization was performed in 96-well plates (Hampton Research, catalog No. HR3-123) that were set up using a Mosquito crystallization robot (TTP Labtech). Prior to crystallization, HSA solution at concentration of 162 mg ml^−1^ (dissolved in 50 m*M* Tris, 20 m*M* NaCl pH 7.4) was mixed with 100 m*M* ketoprofen in 100% DMSO in a 9:1 ratio (final ketoprofen concentration of 10 m*M*) and incubated for several hours at 37°C. Aliquots of 0.2 µl of the resulting HSA–ketoprofen solution were mixed with 0.2 µl aliquots of reservoir solution [50 m*M* potassium phosphate, 24%(*w*/*v*) PEG 3350 pH 7.0]. The crystallization plate was incubated at room temperature for three months and then at 37°C for several days until the first crystals were observed. Harvested crystals were flash-cooled without any additional cryoprotectant.

### 2.4. Data collection and structure determination

Data collection was performed from a single crystal on the SBC 19-ID beamline at the Advanced Photon Source, Argonne National Laboratory, Argonne, Illinois, USA. The experiment was performed at 100 K using X-rays with wavelength 0.979 Å. *HKL*-3000 (Minor *et al.*, 2006 ▸; Otwinowski & Minor, 1997 ▸) was used to process, integrate and scale the data. Corrections for radiation decay and anisotropic diffraction were applied (Borek *et al.*, 2010 ▸). The native structure of HSA (PDB entry 4k2c; Wang, Yu *et al.*, 2013 ▸) was used as the template for molecular replacement. Structure determination and refinement were performed using *HKL*-3000 integrated with *MOLREP* (Vagin & Teplyakov, 2010 ▸), *REFMAC* (Murshudov *et al.*, 2011 ▸), *Coot* (Emsley *et al.*, 2010 ▸) and other programs from the *CCP*4 package (Winn *et al.*, 2011 ▸). The refinement process followed recent state-of-the-art guidelines (Shabalin *et al.*, 2018 ▸; Majorek *et al.*, 2020 ▸). 13 TLS groups determined by the *TLS Motion Determination Server* were applied during refinement (Painter & Merritt, 2006 ▸). (*S*)- or (*R*)-enantiomers of ketoprofen were chosen by careful evaluation of the fit of each candidate to the 2*mF*_o_ − *DF*_c_ and *mF*_o_ − *DF*_c_ omit maps (calculated for ten cycles of *REFMAC* refinement without the ligand). Each choice was supported by comparing the fit to the maps after refinement, the resulting ADP values and the interactions with the protein (hydrogen bonds, salt bridges and lack of clashes). Partial occupancy was evaluated for the (*R*)-ketoprofen molecule in drug site 9, which resulted in the appearance of positive electron density and comparatively low ADP values; therefore, the occupancy was kept at 100%. The *ACHESYM* server (Kowiel *et al.*, 2014 ▸) was used for the standardized placement of the model in the unit cell. The *PISA* server (Krissinel & Henrick, 2007 ▸) was used to analyze the residues involved in interactions between the ligand and macromolecule. *PyMOL* (version 1.5.0.3; Schrödinger) and *ChemSketch* were used for figure generation. The *DALI* server (Holm, 2019 ▸) was used for structure comparison and calculation of C^α^ r.m.s.d. values. The statistics for diffraction data collection, structure refinement and structure quality are summarized in Table 1 ▸. Diffraction images are available at the Integrated Resource for Reproducibility in Macromolecular Crystallography at https://proteindiffraction.org (Grabowski *et al.*, 2016 ▸) with DOI https://doi.org/10.18430/m37jwn. The atomic coordinates and structure factors have been deposited in the PDB with accession code 7jwn.

## 3. Results

### 3.1. Structure of the HSA–ketoprofen complex

**Figure 2:**
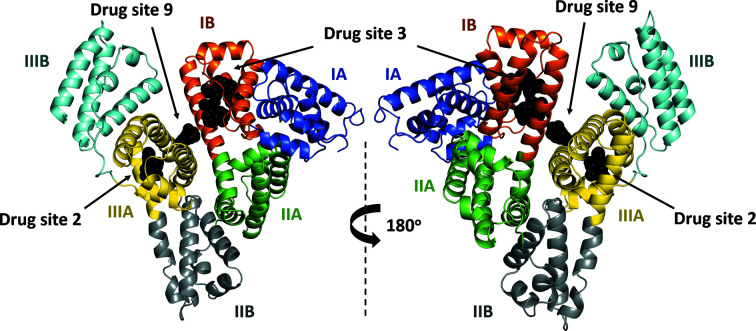
The overall structure of the complex of HSA with ketoprofen. Albumin subdomains are each shown in a different color. Roman numerals (I, II, III) are associated with domains and letters (for example IB) with subdomains. Ketoprofen molecules are shown with atoms in black spheres.

The crystal of the complex of HSA with ketoprofen grew in space group *C*2 and contained one protein chain in the asymmetric unit. The protein model is complete except for the first residue (Ala2), for which electron density was not observed. The electron density revealed the binding of one (*S*)-ketoprofen molecule to drug site 2, two (*S*)-ketoprofen molecules to drug site 3 and one (*R*)-ketoprofen molecule to drug site 9 (Fig. 2 ▸). All three sites were previously reported to bind multiple FDA-approved drugs (Supplementary Fig. S2; Czub *et al.*, 2020 ▸). The structure also contains three fatty-acid molecules, modeled as myristic acid, bound to FA3 (which overlaps with drug site 2), drug site 5 (not previously characterized as a fatty acid-binding site) and FA5 (overlaps with drug site 8). Accordingly, the determined complex of HSA with ketoprofen has an almost identical conformation to HSA complexed with myristic acid (PDB entry 1bj5). However, it is noticeably different from a ligand-free HSA structure (PDB entry 4k2c), as can be concluded from the r.m.s.d. values between the aligned C^α^ atoms (Supplementary Table S1, Supplementary Fig. S3). Fatty acids were not added during crystallization and are most likely remnants from purification. Free cysteine was added by the manufacturer to the protein during purification to block the sole free cysteine residue in HSA and prevent albumin dimerization. Based on the observed electron density, Cys34 forms a disulfide bond with another molecule of cysteine. The quality of electron density observed for ligands in the determined structure can be inspected interactively at https://molstack.bioreproducibility.org/project/view/VW8s7hb1Z9mnCLbg3NBU/. As a control, we also determined a 2.70 Å resolution structure of HSA obtained from the same crystallization conditions but not containing ketoprofen (space group *P*1; unit-cell parameters *a* = 38.0, *b* = 86.2, *c* = 97.3 Å, α = 75.0, β = 89.6, γ = 78.6°; data not shown). In the control structure, all ketoprofen binding sites (drug sites 2, 3 and 9) remain unoccupied, Cys34 also forms a disulfide bond with another molecule of cysteine, and fatty acids bind to the same sites as in the HSA–ketoprofen complex.

### 3.2. Ketoprofen binding sites in HSA

**Figure 3:**
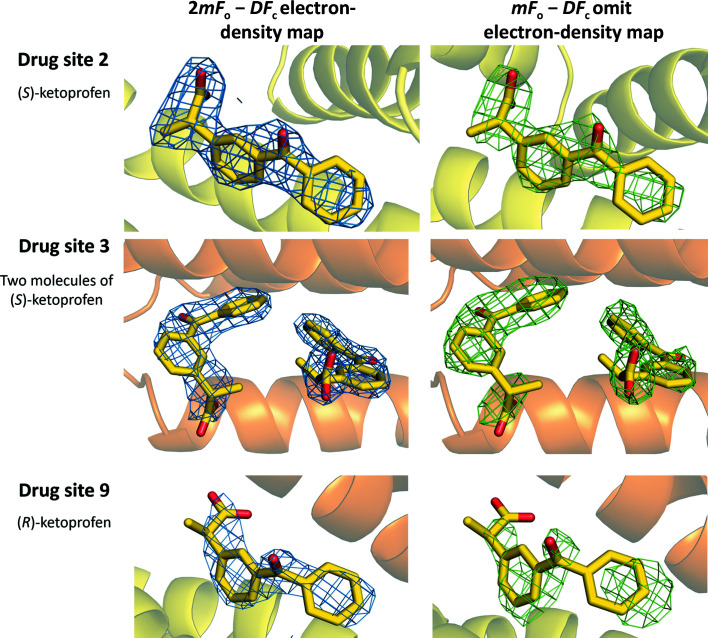
Ketoprofen binding sites in HSA (PDB entry 7jwn). The 2*mF*_o_ − *DF*_c_ electron-density map (r.m.s.d. of 1.0 Å) is presented in blue and the *mF*_o_ − *DF*_c_ omit electron-density map (map calculated after ten *REFMAC* refinement cycles without the drug in the model, r.m.s.d. of 2.5 Å) is presented in green. Ketoprofen molecules are shown in stick representation with O atoms in red and C atoms in yellow. The colors of the helices correspond to the colors used in Fig. 2 ▸. The electron density and the model can be inspected interactively at https://molstack.bioreproducibility.org/project/view/VW8s7hb1Z9mnCLbg3NBU/.

Drug site 2, also known as Sudlow site II and FA3/FA4, is one of the three major drug-binding sites in albumins (Fig. 3 ▸; Czub *et al.*, 2020 ▸; Sudlow *et al.*, 1975 ▸, 1976 ▸). The (*S*)-ketoprofen molecule occupying this site is stabilized by strong hydrophobic interactions with surrounding residues (mainly Tyr411, Val415, Val418, Leu423, Val426, Leu430, Leu453, Val456, Leu457, Leu460 and Phe488) and by hydrogen bonds between its carboxylate group and the hydroxyl groups of Tyr411 and Ser489. Arg410 may also contribute a remote charge–charge interaction with the carboxylate group. The residues involved in the binding of (*S*)-ketoprofen to HSA at drug site 2 are listed in Table 2 ▸. An (*S*)-ketoprofen molecule bound to drug site 2 overlaps with the fatty acid previously reported to bind in FA4 (see PDB entry 1bj5; Curry *et al.*, 1998 ▸) and is located close to FA3, which is occupied by a molecule of myristic acid in the reported structure.

Drug site 3, which is also called the oncological drug site and FA1 (Wang, Ho *et al.*, 2013 ▸), is also one of the three major drug-binding sites on SA (Czub *et al.*, 2020 ▸; Sudlow *et al.*, 1975 ▸, 1976 ▸). This site has two (*S*)-ketoprofen molecules bound and thus can be considered as two subsites. Subsite A overlaps with the previously characterized FA1 site (Curry *et al.*, 1998 ▸) and has (*S*)-ketoprofen bound. (*S*)-Ketoprofen in subsite A is stabilized by strong hydrophobic interactions with residues forming a narrow binding pocket (mainly Leu115, Met123, Phe134, Tyr138, Leu139, Ile142, Leu154, Ala158, Tyr161, Phe165 and Leu182), by a salt bridge between its carboxylate group and the guanidino group of Arg117, and by a hydrogen bond from its carboxylate group to the hydroxyl group of Tyr161 (Table 2 ▸). Moreover, a remote charge–charge interaction of the carboxylate group with Arg186 is likely to be an additional stabilizing factor. Subsite B within drug site 3 harbors an (*S*)-ketoprofen molecule surrounded by sparse hydrophobic residues, mainly Ile142, Phe149, Leu154, Phe157, Tyr161 and the aliphatic part of the side chain of Lys190. At this subsite, the carboxylate group of (*S*)-ketoprofen forms a hydrogen bond to the His146 side chain (NE2 atom) and a remote charge–charge interaction with Arg145. In comparison to drug site 2 and subsite A, subsite B offers a significantly smaller hydrophobicity (as can be seen by the significantly lower number of hydrophobic residues taking part in the interaction) and weaker hydrophilic interactions (no salt bridges and only one hydrogen bond), which may suggest weaker binding of (*S*)-ketoprofen. Indeed, the high atomic ADP values observed for this ligand (Table 1 ▸) may suggest its partial occupancy but may also be a result of its positional variability between HSA molecules in the crystal. Notably, the molecules of (*S*)-ketoprofen bound to the subsites within site 3 have their phenyl rings located within 4 Å of each other (Fig. 3 ▸), suggesting that this hydrophobic interaction additionally stabilizes both (*S*)-ketoprofen molecules and may possibly result in cooperative binding.

Drug site 9, which is located near FA8 and FA9, is a much less common drug-binding site in SA (Czub *et al.*, 2020 ▸). This site contains the only (*R*)-ketoprofen molecule in the reported structure. The (*R*)-ketoprofen molecule is stabilized by several hydrophobic interactions (mainly with Ala191, Ala194, Val433, Tyr452, Val455, Val456 and the aliphatic parts of Lys190 and Lys432); a hydrogen bond is formed between its carboxylate group and the hydroxyl group of Tyr452 and a salt bridge between the carboxylate group and the nitrogen group of Lys436. The (*R*)-ketoprofen molecule has relatively high ADP values, suggesting partial occupancy or positional variability, which is also correlated with a significantly smaller hydrophobicity of this site, which is likely to result in a lower binding affinity.

### 3.3. Comparison of ketoprofen binding sites in HSA and other mammalian SAs

The overall structure of the HSA–ketoprofen complex and the observed individual binding sites were compared with previously reported structures of ESA, BSA and LSA complexed with ketoprofen. A comparison of the experimental conditions that were used and the occupation of the ten established drug-binding sites is summarized in Table 3 ▸.

**Figure 4:**
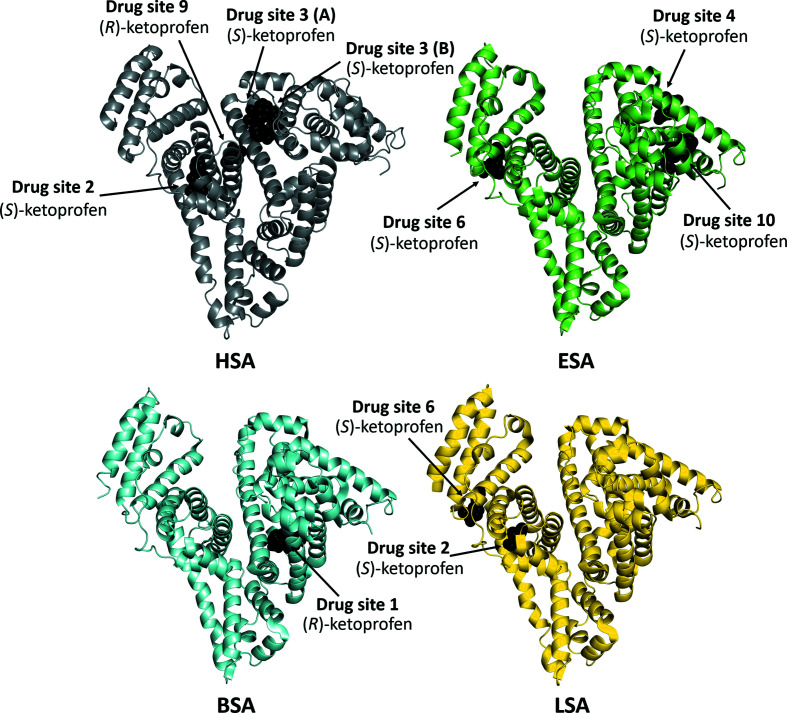
Ketoprofen binding sites in mammalian serum albumins. Structures of ketoprofen complexes with HSA (PDB entry 7jwn), ESA (Czub *et al.*, 2020 ▸; PDB entry 6u4r), BSA (Castagna *et al.*, 2019 ▸; PDB entry 6qs9) and LSA (Zielinski *et al.*, 2020 ▸; PDB entry 6ock).

ESA (pairwise sequence identity of 76.1% to HSA) has recently been reported to bind (*S*)-ketoprofen at drug sites 4, 6 and 10 (Fig. 4 ▸; Czub *et al.*, 2020 ▸). Surprisingly, these drug sites are unoccupied in the HSA structure. The conservation of the residues comprising these drug sites in ESA and HSA has been discussed in detail by Czub *et al.* (2020 ▸), who concluded that drug site 4 differs significantly between ESA and HSA (57% conservation), drug site 6 is partially conserved (75% of residues are conserved) and drug site 10 is very well conserved between albumin from both species (94% conservation). Therefore, the lack of (*S*)-ketoprofen in sites 4 and 6 may be attributed to these differences. However, all of the residues involved in the binding of (*S*)-ketoprofen at drug site 10 in ESA are the same in HSA, except for Ile7, which is a Val in HSA. Moreover, Ile7 in ESA only contributes to hydrophobic interactions with the drug molecule, further suggesting conservation of the site and leading to the expectation that drug site 10 in HSA may also bind (*S*)-ketoprofen, even though it was not observed in the structure reported here. Drug site 2, where (*S*)-ketoprofen binds to HSA, is occupied by a myristate molecule in the structure of the ESA–ketoprofen complex, which potentially prevents drug binding. Drug sites 3 and 9 remain unoccupied in this structure.

**Figure 5:**
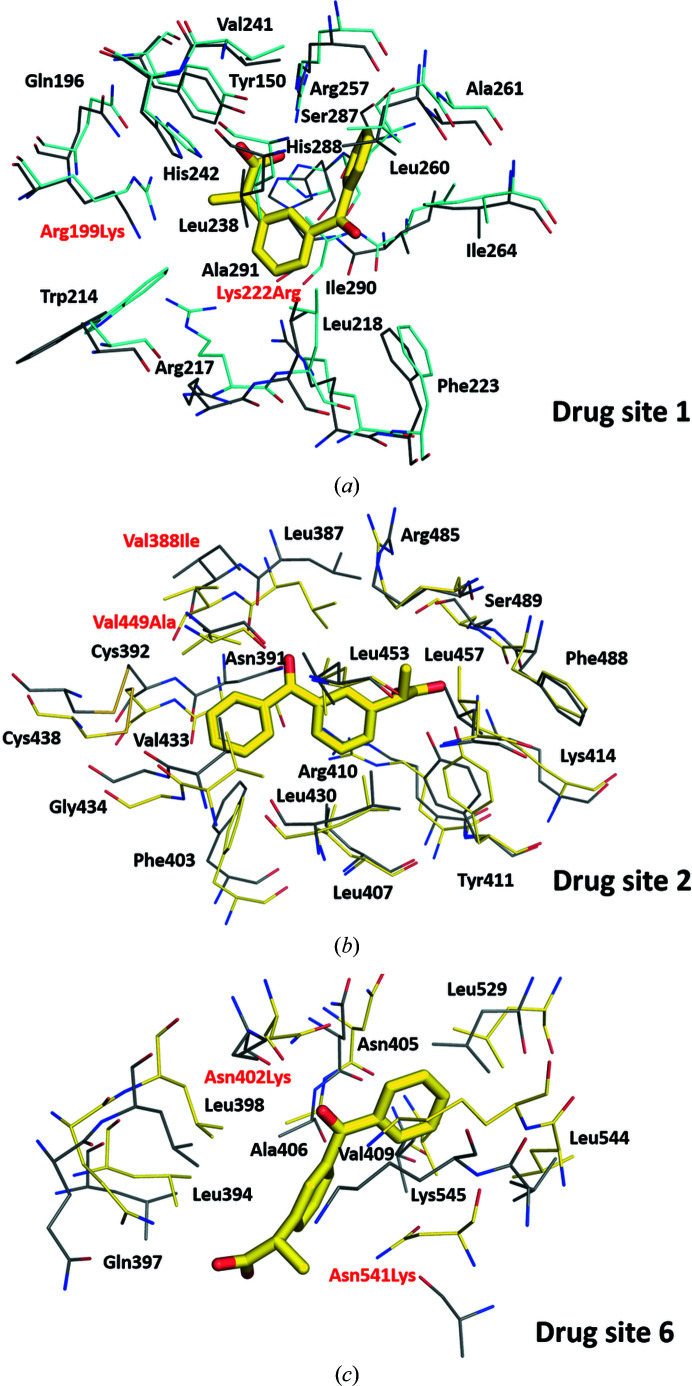
Superposition of ketoprofen binding sites in BSA (*a*) (PDB entry 6qs9) and LSA (*b*, *c*) (PDB entry 6ock) with analogous sites in ligand-free HSA (PDB entry 4k2c). C atoms in BSA, LSA and HSA are shown in cyan, yellow and gray, respectively. Residue numbers correspond to positions in HSA. Residues labeled in black are conserved between BSA or LSA and HSA, while those labeled in red differ. The naming scheme for differing residues is as follows: residue in BSA or LSA, residue number, corresponding residue in HSA.

Drug site 1 (Sudlow site I) has been reported to be the only ketoprofen binding site in BSA (pairwise sequence identity of 75.6% to HSA), with the (*R*)-enantiomer modeled at this site (Fig. 3 ▸; Castagna *et al.*, 2019 ▸). Most of the residues involved in interactions with (*R*)-ketoprofen at drug site 1 are conserved between BSA and HSA (Fig. 5 ▸), including Arg256 (Arg257 in HSA) and Tyr149 (Tyr150 in HSA). These residues form a salt bridge and a hydrogen bond with the carboxylate group of ketoprofen, respectively. Only two residues are different: Arg198 (Lys199 in HSA) and Lys221 (Arg222). Moreover, they are only involved in hydrophobic interactions, and these changes should not affect the binding of ketoprofen to this site. However, despite the very high sequence conservation of drug site 1 between BSA and HSA (89%), this site remains unoccupied in the presented structure of the HSA–ketoprofen complex. Drug sites 2, 3 and 9 are free of ligands in the BSA–ketoprofen structure. The binding of only one ketoprofen molecule to BSA in this structure may be explained by the lower ketoprofen concentration used in comparison to other complexes (Table 3 ▸).

**Figure 6:**
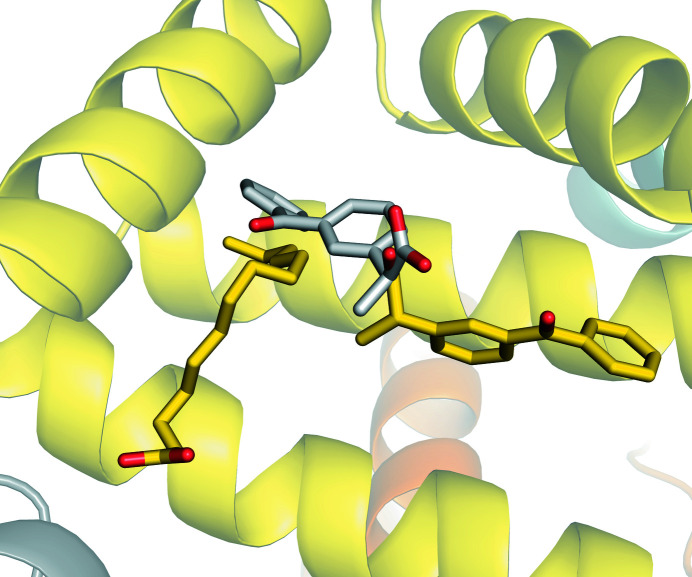
Comparison of (*S*)-ketoprofen binding to drug site 2 in HSA (PDB entry 7jwn) and LSA (PDB entry 6ock). The (*S*)-ketoprofen molecule and a molecule of a fatty acid bound to HSA are shown in stick representation with O atoms in red and C atoms in yellow, while a molecule of (*S*)-ketoprofen bound to LSA is shown in stick representation with O atoms in red and C atoms in gray. The colors of the helices correspond to the colors used in Fig. 2 ▸.

LSA, which has a pairwise sequence identity of 73.4% to HSA, has been reported to bind (*S*)-ketoprofen at drug sites 2 and 6 (Fig. 4 ▸; Zielinski *et al.*, 2020 ▸). Surprisingly, drug site 2 binds (*S*)-ketoprofen in both HSA and LSA, but the binding modes in these structures differ (Fig. 6 ▸). The (*S*)-ketoprofen molecule at drug site 2 in LSA is stabilized by hydrophobic interactions with surrounding residues and forms a salt bridge and a hydrogen bond between its carboxylate group and Lys414 and Tyr411, respectively. Most of the residues involved in these interactions are conserved (89%). In addition, the two residues that differ between LSA and HSA (Val388 is replaced by Ile and Val449 by Ala) do not change the character of the binding site (Fig. 5 ▸). The carboxylate group of the drug occupies roughly the same position, but its hydrophobic moieties are oriented in opposite directions. It is likely that the presence of the myristate molecule at this site (FA3) in the HSA structure reported here affects the conformation of (*S*)-ketoprofen. Previously, (*S*)-ibuprofen was reported to bind to drug site 2 in HSA and ESA via two different respective binding modes that resemble those of (*S*)-ketoprofen (Supplementary Fig. S4; Czub *et al.*, 2020 ▸).

Another (*S*)-ketoprofen molecule binds to drug site 6 in LSA, where it is stabilized by hydrophobic interactions and by hydrogen bonds between its carboxylate group and the side chain of Asn397 (NE2 atom) and between its carbonyl group and the side chains of Asn402 (ND2 atom) and Lys545. This binding site is 82% conserved between LSA and HSA. Two residues that differ in HSA (Asn402 to Lys and Asn541 to Lys) change the overall charge of the cavity, and due to the larger size of the Lys side chains in HSA may affect the conformation of the drug or even prevent binding completely. Drug site 6 is unoccupied in the HSA–ketoprofen structure. In the LSA–ketoprofen structure, drug site 3 is occupied by a polyethylene glycol molecule, which may prevent drug binding to this site. An acetate ion is present in drug site 9.

## 4. Discussion

The structure of the complex of ketoprofen with HSA revealed that four ketoprofen molecules bind to drug sites 2, 3 and 9. The electron-density map of the ketoprofen molecules indicated the binding of (*S*)-enantiomers at drug sites 2 and 3 (two molecules) and an (*R*)-enantiomer at drug site 9. We compared the HSA–ketoprofen complex with previously reported ketoprofen–albumin complexes from other mammals. Ketoprofen was shown to bind to drug sites 4, 6 and 10 in ESA (Czub *et al.*, 2020 ▸), drug site 1 in BSA (Castagna *et al.*, 2019 ▸) and drug sites 2 and 6 in LSA (Fig. 4 ▸; Zielinski *et al.*, 2020 ▸). Despite high sequence identity (the pairwise sequence identity to HSA is 76.1% for ESA, 75.6% for BSA and 73.4% for LSA; Supplementary Fig. S1), well conserved binding sites (Fig. 5 ▸) and similar ketoprofen binding affinities (Bi *et al.*, 2011 ▸; Zielinski *et al.*, 2020 ▸; Misra & Kishore, 2013 ▸), drug site 2 is the only site that was observed to be occupied by ketoprofen in HSA and an albumin from another species (LSA). The residues involved in ketoprofen binding to drug site 2 are 89% conserved between LSA and HSA, and only two of them differ (in LSA Ile388 is replaced by Val and Ala449 by Val). These modified residues contribute to hydrophobic inter­actions and their alterations represent very conservative changes to the character of this site. However, the structures discussed here show that the albumin drug sites to which a particular drug binds cannot be easily predicted based only on a known complex of albumin from another organism and the conservation of drug sites between species. For instance, drug site 10 is very well conserved between ESA and HSA (94% of residues are conserved), including its character and protein fold, but ketoprofen binding to this site was only observed for ESA. Therefore, it appears likely that very subtle differences in amino-acid residues at each site are sufficient to alter the binding-site preference. This would mean that the usefulness of nonhuman albumins and animal models in studies of HSA–drug interactions, drug efficacy and drug pharmacokinetics is limited.

The variations in ketoprofen binding-site preference observed across albumins from different organisms may also depend on crystallization conditions (Table 3 ▸). In the structure of LSA, some of the binding sites are occupied by compounds used as part of the crystallization conditions (for example, drug site 3 is occupied by a molecule of polyethylene glycol). Moreover, the ESA–ketoprofen complex is the only complex that was crystallized from a high-salt condition without PEGs present. All other complexes discussed here were obtained from conditions with 16–24% PEG and with much lower salt concentrations. It is possible that these and other differences (for example the presence of different organic solvents used to dissolve the drugs) contribute to the differences in occupied sites observed, as ionic strength, viscosity and temperature have been shown to affect ligand binding to proteins (Papaneophytou *et al.*, 2014 ▸). Another potential contributing factor to the variations in binding-site specificity are the compounds present in the protein preparations. In the HSA and ESA structures, some drug sites are occupied by fatty acids that were not added during crystallization (see Table 3 ▸). For instance, drug site 2 in the ESA–ketoprofen complex is occupied by a fatty-acid molecule, potentially preventing drug binding. In addition to the direct competition from fatty acids for drug-binding sites, fatty acids also change the overall conformation of albumin and affect the affinity of drugs at specific binding sites (Curry *et al.*, 1998 ▸; Petitpas *et al.*, 2003 ▸). For example, HSA in the determined structure has a conformation significantly different from HSA without bound fatty acids and is different from other albumin complexes with ketoprofen bound (Supplementary Fig. S3). The calculated r.m.s.d. values are higher in the structural comparison of complexes with fatty acids and ligand-free albumins than when comparing albumin complexes with ketoprofen and ligand-free albumins (Supplementary Table S1). These results indicate that fatty-acid binding alters the conformation of albumin more significantly than the binding of ketoprofen. Plasma fatty-acid levels are dynamic and are chronically elevated in some disease states (Sobczak *et al.*, 2019 ▸; Boden, 2011 ▸; Li *et al.*, 2018 ▸), which affects the binding of drugs and other molecules (Ghuman *et al.*, 2005 ▸; Dobretsov *et al.*, 2012 ▸). Similarly, other metabolites that interact with albumin under physiological conditions (for example glucose) can occupy drug sites and even chemically modify the albumin structure, as in the case of non-enzymatic glycosylation (Anguizola *et al.*, 2013 ▸). Subsequently, albumin isolated from natural sources may have different impurities or different chemical modifications to recombinantly expressed albumin. These modifications are likely to affect a small percentage of albumin molecules and, as a result, are not usually observed in crystal structures, with the exception here being fatty acids. However, variations in plasma composition among organisms, as well as individuals of the same species, can contribute to differences in the drug affinity measured for HSA and albumin from other species *in vitro*. Another important factor that affects the number of binding sites observed for a particular drug is the concentration of the drug used in crystallization or crystal soaking. It is known that drug sites on albumin have different affinities for ligands, and ketoprofen could bind to lower-affinity sites at a sufficiently high occupancy to be seen in the electron-density maps when a high concentration of the drug is used. Additionally, the way that the ligand was added to the protein, namely co-crystallization or crystal soaking, can affect where it binds to albumin due to potential differences in site accessibility in the solution *versus* the crystal form. Due to all of the abovementioned factors, it is of course not possible to carry out experiments that control for all potential variables or indeed to measure the relative affinities at each binding site.

HSA is known to bind chiral drugs stereoselectively (Shen *et al.*, 2013 ▸). In the reported structure of HSA, we observed the binding of three (*S*)-ketoprofen molecules (drug sites 2 and 3) and only one (*R*)-ketoprofen molecule (drug site 9). The shape of drug sites 2, 3 and 9 clearly supports the binding of the specific enantiomer of ketoprofen, suggesting differences in the binding affinity of HSA to the respective ketoprofen enantiomers. This observation agrees with previous reports stating that HSA can bind (*R*)- and (*S*)-enantiomers of ketoprofen with different affinities depending on the experimental conditions (Dubois *et al.*, 1993 ▸; Zhivkova & Russeva, 1998 ▸). We can also conclude that albumin typically promotes the binding of (*S*)-‘profens’, but its stereoselectivity is ultimately dictated by the structure of the drug and its fit to a particular binding site. The ten binding sites available on albumins have differing affinities for different stereoisomers, and some of them may go against the general trend, as is in the case of site 9, which binds (*R*)-ketoprofen. We believe that different stereoisomers of drugs have distinct binding affinities to various SA drug-binding sites simply because they differ in spatial structure and their binding is affected by shape complementarity.

The results reported here provide insight into the molecular basis of ketoprofen transport across species and indicate a need for similar studies for other drugs. Structural determination and biochemical characterization of drug complexes relating to HSA in parallel with those involving albumin from animals used as model organisms, are necessary to evaluate the appropriateness of such models and contribute to our understanding of drug transport. In cases of high sequence similarity, the conservation of a particular ligand–protein interaction across organisms is traditionally expected. However, this study shows that this assumption is not necessarily valid, at least for ketoprofen–albumin complex studies. It remains to be seen whether these differences in drug binding to albumin from different species will be discovered for other drugs.

## Supplementary Material

PDB reference: human serum albumin, complex with ketoprofen, 7jwn

Supplementary Figures and Tables. DOI: 10.1107/S2052252522006820/lz5056sup1.pdf

Diffraction images.: https://doi.org/10.18430/m37jwn

A preprint of this article is available from bioRxiv.: https://doi.org/10.1101/2021.04.03.438117

## Figures and Tables

**Table 1:** Data-collection, structure-refinement and structure-quality statistics Values in parentheses are for the highest resolution shell. Ramachandran plot statistics are calculated by *MolProbity* (Williams *et al.*, 2018 ▸). DS2, DS3 and DS9 refer to drug-binding sites 2, 3 and 9, respectively.

PDB code	7jwn
Diffraction images DOI	https://doi.org/10.18430/m37jwn
Resolution (Å)	50.00–2.60 (2.64–2.60)
Wavelength (Å)	0.979
Space group	*C*2
*a*, *b*, *c* (Å)	170.5, 38.9, 98.5
α, β, γ (°)	90.0, 104.5, 90.0
Protein chains in the asymmetric unit	1
Completeness (%)	96.4 (88.5)
No. of unique reflections	18925 (851)
Multiplicity	4.2 (3.5)
〈*I*〉/〈σ(*I*)〉	16.9 (1.3)
CC_1/2_	(0.60)
*R*_merge_	0.081 (0.803)
*R*_meas_	0.093 (0.925)
*R*_work_/*R*_free_	0.183/0.231
R.m.s.d, bond lengths (Å)	0.002
R.m.s.d, bond angles (°)	1.1
Mean ADP (Å^2^)	52
Mean ADP for ketoprofen molecules (Å^2^)	
(*S*)-Ketoprofen	24.4 [DS2], 16.6 [DS3, subsite A], 47.1 [DS3, subsite B]
(*R*)-Ketoprofen	72.1 [DS9]
No. of protein atoms	4646
Mean ADP for protein (Å^2^)	53
No. of water molecules	192
Mean ADP for water molecules (Å^2^)	36
Clashscore	1.27
*MolProbity* score	1.07
Rotamer outliers (%)	0.59
Ramachandran outliers (%)	0.0
Ramachandran favored (%)	96.23

**Table 2:** The residues that participate in the binding of ketoprofen to HSA and the hydrophilic interactions observed in ketoprofen binding sites Residues shown in bold provide a major hydrophobic contribution to drug binding.

Drug site	Subdomains	Drug	Residues	Salt bridges and hydrogen bonds
2	IIIA	(*S*)-Ketoprofen	Arg410, **Tyr411**, Lys414, **Val415**, **Val418**, **Leu423**, **Val426**, **Leu430**, **Leu453**, **Val456**, **Leu457**, **Leu460**, Val473, Arg485, **Phe488**, Ser489, Leu491	The carboxylate group of (*S*)-ketoprofen forms hydrogen bonds to the hydroxyl groups of Tyr411 and Ser489 and a remote charge–charge inter­action with Arg410
3, subsite A	IB	(*S*)-Ketoprofen	**Leu115**, Arg117, **Met123**, **Phe134**, Leu135, **Tyr138**, **Leu139**, **Ile142**, **Leu154**, Phe157, **Ala158**, **Tyr161**, **Phe165**, **Leu182**, Arg186	The carboxylate group of (*S*)-ketoprofen forms a salt bridge with the guanidino group of Arg117, a hydrogen bond to the hydroxyl group of Tyr161 and a remote charge–charge interaction with Arg186
3, subsite B		(*S*)-Ketoprofen	Leu115, **Ile142**, Arg145, His146, **Phe149**, **Leu154**, **Phe157**, **Tyr161**, Leu185, Arg186, Gly189, **Lys190**, Ser193	The carboxylate group of (*S*)-ketoprofen forms a hydrogen bond to the side chain of His146 (NE2 atom) and a remote charge–charge interaction with Arg145
9	IB and IIIA	(*R*)-Ketoprofen	Glu184, Asp187, Glu188, **Lys190**, **Ala191**, **Ala194**, Glu425, Asn429, **Lys432**, **Val433**, Lys436, **Tyr452**, **Val455**, **Val456**, Gln459	The carboxylate group of (*R*)-ketoprofen forms a salt bridge with the nitrogen group of Lys436 and a hydrogen bond to the hydroxyl group of Tyr452

**Table 3:** Conditions used for crystallization of SA–ketoprofen complexes and the ligands observed in the common drug-binding sites

	HSA–ketoprofen	ESA–ketoprofen	BSA–ketoprofen	LSA–ketoprofen
PDB code	7jwn	6u4r	6qs9	6ock
Reference	This work	Czub *et al.* (2020 ▸)	Castagna *et al.* (2019 ▸)	Zielinski *et al.* (2020 ▸)
SA source	Recombinant HSA expressed in *P. pastoris* (Sigma A7736)	ESA isolated from horse blood (Equitech-Bio ESA62)	BSA isolated from bovine blood (Sigma)	LSA isolated from leporine blood (Sigma) and defatted prior to the experiment
Crystallization drops	An HSA (162 mg ml^−1^) buffered solution (50 m*M* Tris, 20 m*M* NaCl pH 7.5) was mixed with 100 m*M* ketoprofen in DMSO in a 9:1 ratio. The HSA solution was mixed 1:1 with the reservoir solution.	An ESA (34 mg ml^−1^) buffered solution (10 m*M* Tris, 150 m*M* NaCl pH 7.5) was mixed 1:1 with the reservoir solution. ESA crystals were soaked with ketoprofen suspended in DMSO.	A BSA (10 mg ml^−1^) buffered solution (10 m*M* Tris, 150 m*M* NaCl pH 7.5) was mixed with ketoprofen dissolved in ethanol. The BSA solution was mixed 1:1 with the reservoir solution.	An LSA (67 mg ml^−1^) buffered solution (10 m*M* Tris, 100 m*M* NaCl pH 7.4) was mixed with ketoprofen dissolved in ethanol. The LSA solution was mixed 1:1 with the reservoir solution.
Final ketoprofen concentration (m*M*)	5.0	3.0	0.7	4.6
Reservoir solution	50 m*M* potassium phosphate, 24% PEG 3350 pH 7.0	100 m*M* Tris, 2.0 *M* ammonium sulfate, 200 m*M* lithium sulfate pH 7.4	100 m*M* MES, 18% PEG MME 5000, 200 m*M* ammonium chloride pH 6.5	100 m*M* Tris, 8% polypropylene glycol 400, 16% PEG 3350, 200 m*M* ammonium acetate pH 8.0
DS1	—	UNL	(*R*)-Ketoprofen	Acetate ion
DS2	(*S*)-Ketoprofen, fatty acid (C14:0; myristate)	Fatty acid (C9:0; nonanoic acid)	—	(*S*)-Ketoprofen
DS3	Two molecules of (*S*)-ketoprofen	—	—	PEG molecule
DS4	—	(*S*)-Ketoprofen	—	Polymer with PDB code 2J3
DS5	Fatty acid (C14:0; myristate)	—	—	—
DS6	—	(*S*)-Ketoprofen	—	(*S*)-Ketoprofen, acetate ion
DS7	—	—	—	Polymer with PDB code POG
DS8	Fatty acid (C14:0; myristate)	—	—	—
DS9	(*R*)-Ketoprofen	—	—	Acetate ion
DS10	—	(*S*)-Ketoprofen	—	—
